# Efficient digital design of the nonlinear behavior of Hindmarsh–Rose neuron model in large-scale neural population

**DOI:** 10.1038/s41598-024-54525-8

**Published:** 2024-02-15

**Authors:** Soheila Nazari, Shabnam Jamshidi

**Affiliations:** Faculty of Electrical Engineering, Shahid Beheshti University, Tehran, Iran

**Keywords:** Hindmarsh–Rose (HR) neuron, CORDIC_HR model, Digital design, Spiking frequency gate based on CORDIC_HR, Spiking image processing, Computational biology and bioinformatics, Engineering, Mathematics and computing

## Abstract

Spiking networks, as the third generation of neural networks, are of great interest today due to their low power consumption in cognitive processes. This important characteristic has caused the hardware implementation techniques of spiking networks in the form of neuromorphic systems attract a lot of attention. For the first time, the focus is on the digital implementation based on CORDIC approximation of the Hindmarsh–Rose (HR) neuron so that the hardware implementation cost is lower than previous studies. If the digital design of a neuron is done efficient, the possibility of implementing a population of neurons is provided for the feasibility of low-consumption implementation of high-level cognitive processes in hardware, which is considered in this paper through edge detector, noise removal and image magnification spiking networks based on the proposed CORDIC_HR model. While using less hardware resources, the proposed HR neuron model follows the behavior of the original neuron model in the time domain with much less error than previous study. Also, the complex nonlinear behavior of the original and the proposed model of HR neuron through the bifurcation diagram, phase space and nullcline space analysis under different system parameters was investigated and the good follow-up of the proposed model was confirmed from the original model. In addition to the fact that the individual behavior of the original and the proposed neurons is the same, the functional and behavioral performance of the randomly connected neuronal population of original and proposed neuron model is equal. In general, the main contribution of the paper is in presenting an efficient hardware model, which consumes less hardware resources, follows the behavior of the original model with high accuracy, and has an acceptable performance in image processing applications such as noise removal and edge detection.

## Introduction

The perceptron or McCulloch–Pitts neuron was the first neural network^1^. Second-generation neural networks comprise of neurons that apply non-linear activation functions to the sum of the weighted inputs, producing a continuous value output^1^. Neurons have been identified as the most important part in the vast world of biological components of the nervous system^2^. Neurons are connected through excitatory and inhibitory synapses and form the dominant interactions of the nervous system^2^. Of course, in this complex system, there are also glial cells that play a supporting role for neurons and are connected to each other through gap junctions^3^. Spiking neural networks (SNNs) as the third generation of neural networks was proposed to better emulate real biological nervous systems through spike-based information encoding and transmission^4^.

Despites the high number of neurons and biological elements and many synaptic connections, the human nervous system consumes 10 to 20 W of power even in high-level cognitive processes^5^. The unique characteristics of the low power consumption of the nervous system is something that today’s intelligence machines do not have, and spiking networks, by adapting the neural system calculations, aim to achieve this^6–8^. In fact, spiking networks have much lower power consumption than second and first generations neural networks due to their event-based and asynchronous nature so that spiking Yolo network consumes almost 280 times less energy than deep Yolo^7^. Therefore, the hardware implementation of spiking networks is of great importance.

Nevertheless, the efficient hardware design of neurons^9^, astrocytes^10,11^, and synapses^9^ as the main components of the spiking neural networks is important. Considering the limitation of hardware resources and the size of the designed electronic chips, the efficient hardware implementation of neurons provides the possibility of creating a large-scale neural population on the chip^12^. Also, with efficient hardware implementation of neural synapses, the possibility of transmitting spikes with low power consumption is provided^13^. In this paper, it is focused on the hardware implementation of the biological Hindmarsh–Rose (HR) neuron model, and the complex and dynamic behaviors of the designed digital neuron have matched well with the original neuron model.

Due to the significant importance of developing systems with spike calculations, in recent years, many studies have focused on application of computational models of neurons, including leaky integrate and fire neuron (LIF)^14^, Izhikevich^15^, Hindmarsh–Rose (HR)^16^, Morris–Lecar^17^, Hodgkin–Huxley (HH) neuron model^18^. Some of computational models of neurons, such as LIF, have low biological richness and computational cost, and some of them, such as HH, have high biological richness and computational cost. The HR neuron model has a high biological richness among the presented neuron models and has a lower computational cost compared to HH neuron, which has the highest biological richness.

Hardware implementation of neural models is possible in three ways: digital design, analog design, and mixed mode analog/digital. Although analog implementation is more efficient, due to the time-consuming design process, influence of noise, and inflexibility has a lower priority than digital design^19^. Among the digital platforms for hardware implementation, FPGA (Field-Programmable Gate Array) has been able to attract more attention in the applications of neuromorphic system design^20^. FPGAs are a good choice for the application considered in this paper due to their flexibility, availability, and providing a scalable resource of digital gates for the development of large-scale spiking networks.

In the field of digital implementation of neuromorphic systems on FPGA, many studies have been published. In second generation neural networks and deep networks, the neuron appears in the form of an activation function, and the activation function must be approximated for efficient digital implementation^21^, but in spiking networks, the neuron appears in the form of a dynamic model which nonlinear terms should be simplified to reduce computational cost of digital implementation. Various techniques have been used in the FPGA implementation of spiking neural models, among which techniques nonlinear approximation based on LUT^22^, piecewise linear^23^, Single Constant Multiply (SCM)^9^, and Coordinate Rotation Digital Computer (CORDIC)^24^ can be mentioned.

According to the studied background in the digital implementation of different neuron models, the importance of the efficient implementation of the biological neuron as a part of neuromorphic systems which make possible implementation of the spiking networks with cognitive application on the electronic chip is evident. In this regard, we have focused on the digital implementation of the HR neuron model, which has three differential equations with the ability to generate all types of spikes and bursts behaviors. Non-linear terms such as power 2 and power 3 in the differential equations of HR model are performed using shift and addition operations based on the proposed CORDIC module (CORDIC_Pow_2, CORDIC_Pow_3) and multiplier less digital implementation of this nonlinear neuron model on FPGA has been provided. The CORDIC based model of HR neuron is an approximate model of the original HR neuron model, which called CORDIC_HR neuron model. CORDIC_HR compared to HR neuron model require less resource utilization, less area and has higher speed, and consequently lower power consumption. The efficient digital design of the CORDIC_HR neuron model, which consumes less resources than previous studies, provide low-cost implementation of large-scale neuronal population on hardware.

While the proposed CORDIC_HR model requires much less hardware resources than the original HR neuron model, it completely follows the behavior of the original model in terms of dynamic behavior. To ensure that the performance of the proposed CORDIC_HR model matches the original model, the spiking and burst response in the time domain, the behavior of the phase space, the bifurcation diagram, and the movement of the trajectories in the nullcline space using the CORDIC_HR and original HR model are compared and the exact performance of the proposed CORDIC_HR model was approved.

In addition, in comparing the behavior of the CORDIC_HR and HR model, not only the behavior of a single neuron should be considered, but the behavior of the CORDIC_HR model in a network of proposed neurons should be the same compared to the original model. Therefore, a population of 1000 randomly connected CORDIC_HR neurons is designed and its behavior is completely consistent with a population of 1000 HR neurons. On the other hand, to ensure the accuracy of the CORDIC_HR’s performance in cognitive functions, the CORDIC_HR model has been used in the design of spiking frequency gates (SFGs) and consequently spiking image processing unit, and the results of this stage also confirm the accurate performance of the proposed CORDIC_HR model.

The rest of paper is organized as follows.

The computational model of HR and CORDIC_HR is discussed in “Computational model of HR neuron model” and “CORDIC_HR neuron model” sections. Large scale simulation of CORDIC_HR neurons is considered in the “Large scale network of CRDIC_HR model” section. The hardware implementation and discussion are placed in “Digital circuit design” and “Discussion” sections and finally “Conclusion” section concludes the paper.

## Computational model of HR neuron model

In 1984, Hindmarsh and Rose presented a simplified model of the Hodgkin-Huxley (HH) neuron under the title Hindmarsh–Rose (HR) neuron^25^. Neuron HR is a three-dimensional model and can produce all types of dynamic behaviors of a biological neuron, so that it can accurately models current and voltage oscillations in the membrane of the nerve fiber. Therefore, a detailed and complete analysis of the dynamic behavior of this computational model can provide a comprehensive comprehension of the characteristics of the biological system, which can be effective in exploring biological mechanisms^26^. The mathematical equations of the HR neuron are defined as follows:

1 $$\left\{\begin{array}{l}\frac{dX}{dt}=Y+{F}_{NL}\left(X\right)\left[3-X\right]-Z+I\\ \frac{dY}{dt}=1-5{F}_{NL}\left(X\right)-Y\\ \frac{dZ}{dt}=r(H\left(X\right)-Z)\end{array},\right.$$

2 $$\left\{\begin{array}{l}{F}_{NL}\left(X\right)={X}^{2}\\ H\left(X\right)=4[X+1.6]\end{array}\right..$$

Membrane potential, fast current corresponding to sodium and potassium ion channel dynamics, slow current corresponding to calcium channel dynamics are indicated by X, Y and Z, respectively. Also, *I* is the input stimulation current and *r* is the spike frequency controller, which by changing these two parameters, all kinds of spike and burst behaviors (tonic and periodic) and chaotic behavior are produced, some of which are shown in Fig. 5. As it is evident in the equations, the three-dimensional differential equations of HR include the nonlinear function ${F}_{NL}\left(X\right)$, which creates the nonlinear terms ${X}^{2}$ and ${X}^{3}$. Non-linear terms and the use of multipliers increase the hardware implementation cost and challenge the possibility of large-scale neural network implementation^9^. To deal with this problem, CORDIC_HR model by replacing the nonlinear terms of HR model with efficiently designed CORDIC blocks has been presented. The CORDIC_HR model make possible to implement low-cost hardware, while the dynamic characteristics of the original model are completely preserved.

## CORDIC_HR neuron model

Because of having a compact circuit with minimum resources consumption and maximum speed, the nonlinear terms of HR neuron model (${X}^{2}, {X}^{3}$) must be simplified. In this paper, the simplification of the nonlinear terms of the HR neuron has been done using the CORDIC algorithm without multipliers. Using CORDIC_POW_2 and CORDIC_POW_3 blocks instead of terms ${X}^{2}, {X}^{3}$, an approximate model of neuron HR is presented, which is named as CORDIC_HR neuron, and its relation are according to the Eq. (3).

3 $$\left\{\begin{array}{l}\frac{dX}{dt}=Y+3\times CORDIC\_POW\_2(X)-CORDIC\_POW\_3(X)-Z+I\\ \frac{dY}{dt}=1-5\times CORDIC\_POW\_2(X)-Y\\ \frac{dZ}{dt}=4r\times X+6.4\times r-r\times Z\end{array}.\right.$$

Compared to previous works, the proposed CORDIC_HR model has the most compatibility with the original model (HR neuron) and at the same time consumes the least resources in hardware implementation. To introduce CORDIC_POW_2 and CORDIC_POW_3 blocks, first the CORDIC multiplier block is introduced.

**Figure 1:**
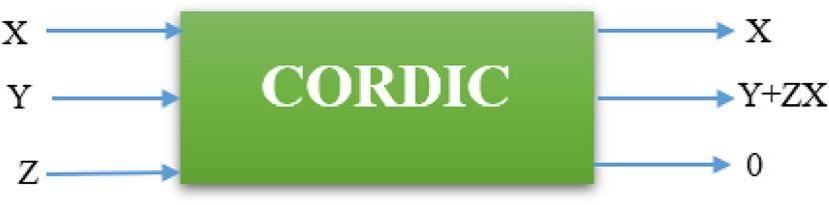
CORDIC algorithm in the linear mode.

To have a multiplication operation, we must use the linear mode in the rotation mode of the CORDIC algorithm. According to Fig. 1, if the initial *Y* is equal to zero and the initial *Z* is equal to *X*, then *Y* will be equal to ${X}^{2}$.

**Figure 2:**
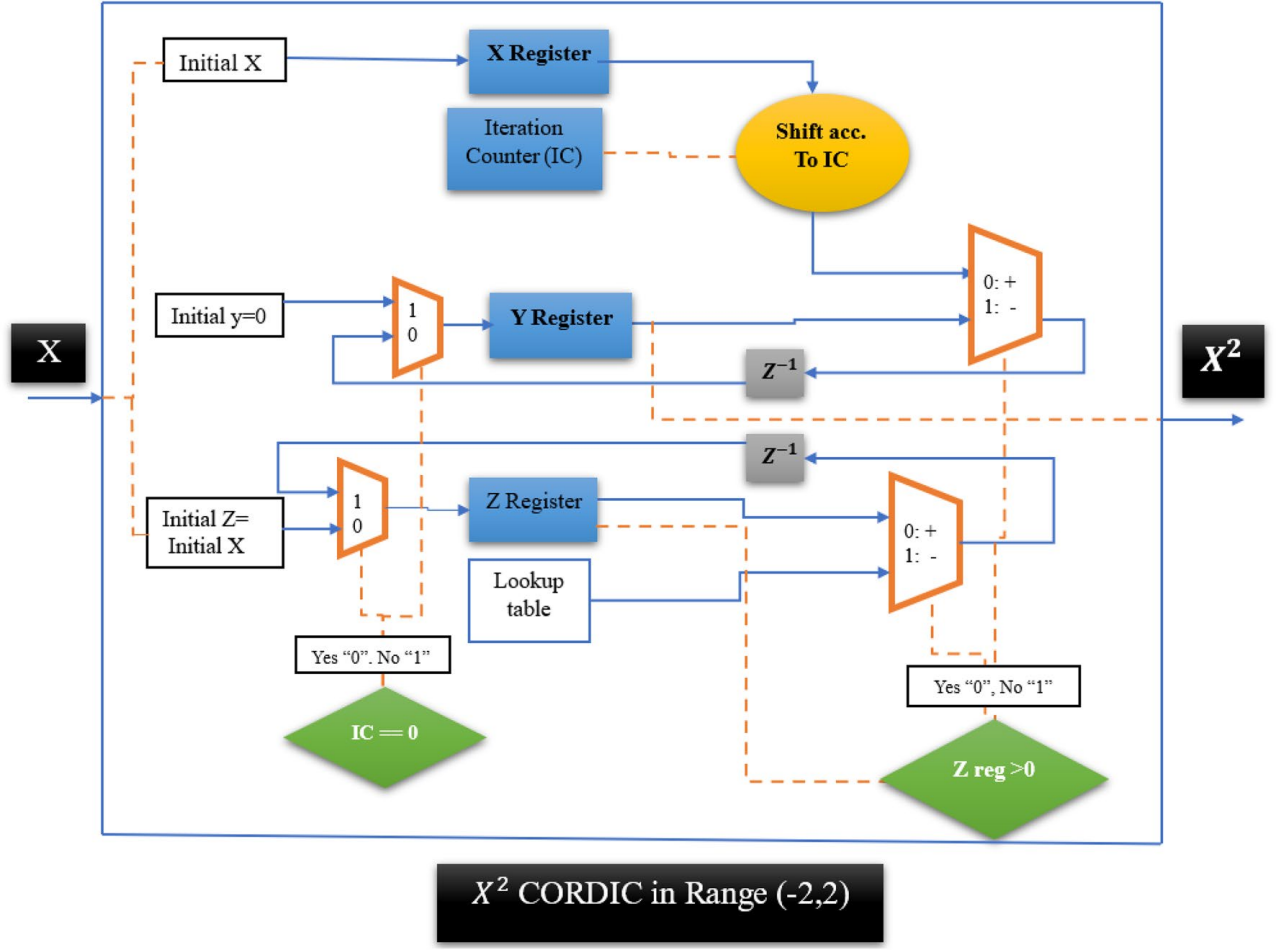
CORDIC power of 2 which is defined in the range − 2 to 2.

As shown in Fig. 1, *Z* starts to change towards zero from the initial value of *X*. Also, *X* remains constant and *Y* changes from zero to the value $XZ={X}^{2}$. Each CORDIC_POW_2 block has two inputs and one output. Two inputs are equal to *X* and the output is equal to the product of two inputs i.e. ${X}^{2}$. Figure 2 shows the block of CORDIC_POW_2 in the range − 2 to 2.

**Figure 3:**
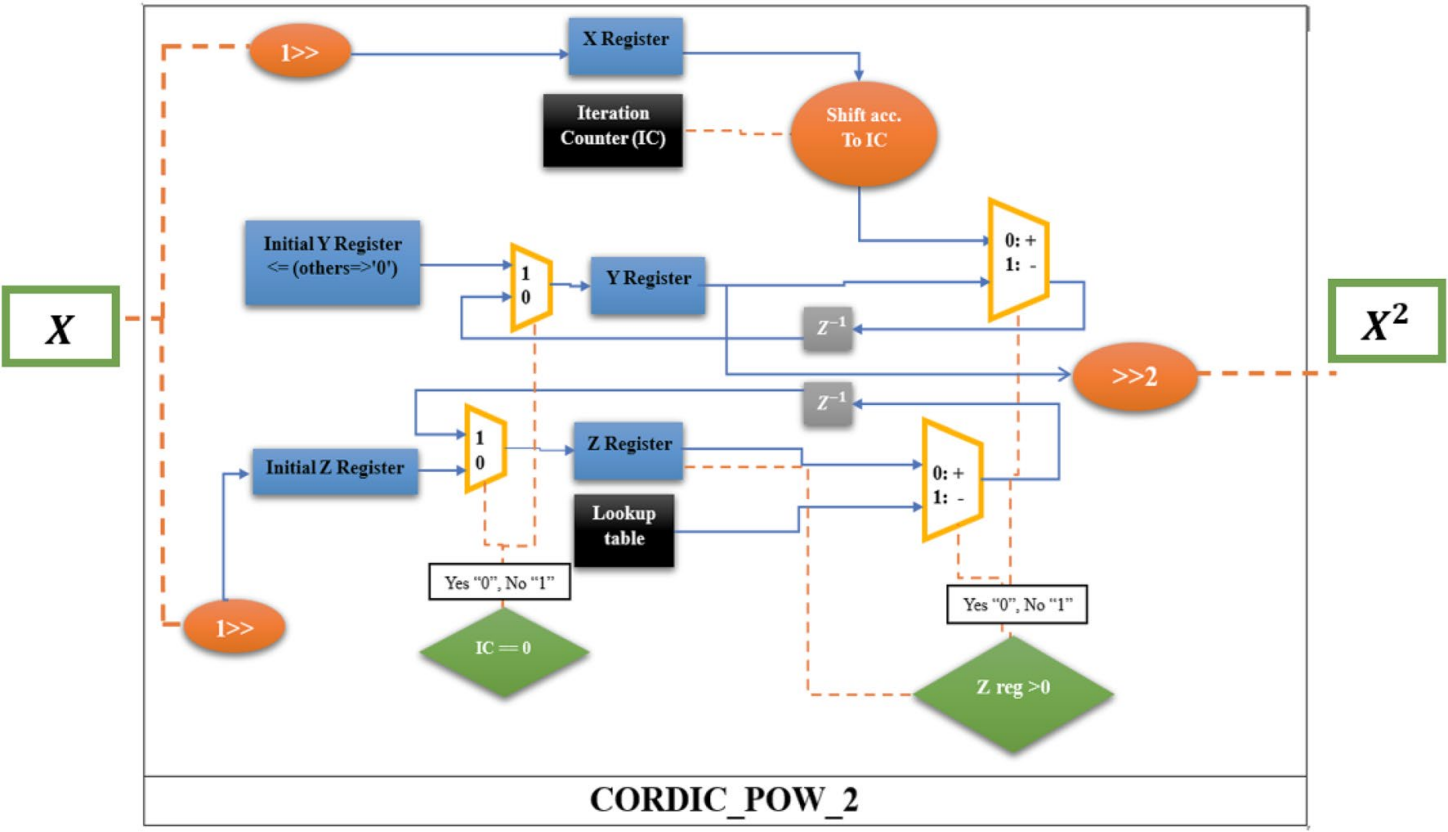
CORDIC block of the power of two which is defined in the interval (− 2, 2.5).

Considering that *X* in the HR neuron equations changes in the range of − 2 to 2.5, so the CORDIC block of power 2 in Fig. 2 is modified to Fig. 3.

The CORDIC_Pow_2 block in Fig. 3 is acceptable for the interval (− 2, 2.5). The technique used for mapping input range (− 2, 2) to (−  2, 2.5) is to first divide the input by 2 (with shift), then give it to the CORDIC power module in Fig. 2, then multiply the output by 4 (with shift) as indicated in Fig. 3.

**Figure 4:**
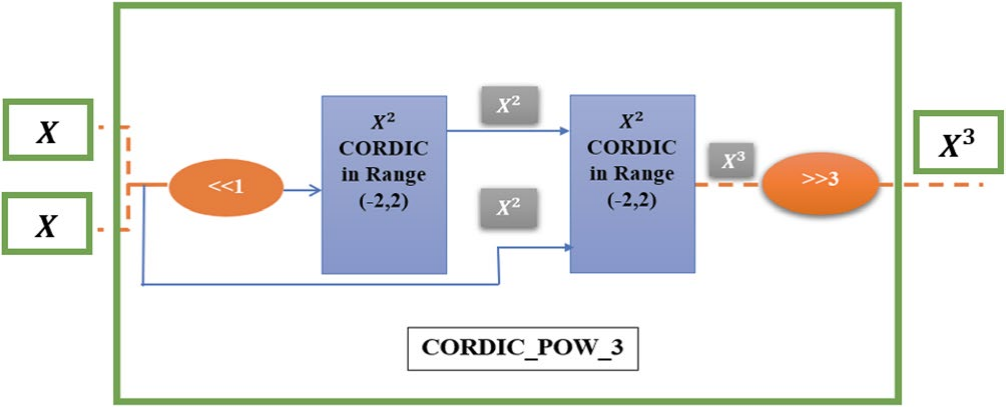
CORDIC_POW_3, this block consists of two ${X}^{2}$ CORDIC blocks in the interval (− 2, 2).

The CORDIC block of the power of 3 with the help of two ${X}^{2}$ CORDIC modules in the range (− 2, 2) can be calculated, which is shown in Fig. 4.

The third power of *X* must be in the range of changes of variable *X* in the equations of HR neuron. Thus, the first and last shifts in the designed block of Fig. 4 are considered.

The proposed CORDIC_HR model should be able to closely follow the HR neuron model. For this purpose, in the validation procedure of the proposed model, spiking response, dynamic behavior in nullcline space, phase space behavior and the bifurcation diagram of the CORDIC_HR model compared to the HR model have been investigated.

## Validation of CORDIC_HR model

In this section, the compatibility of two CORDIC_HR and HR models in the responses of the time domain, nullcline space and how attractors are attracted and rejected, phase space and bifurcation diagram have been investigated respectively.

### Investigating the time domain behavior of the CORDIC_HR model

To check the correspondence of the time domain behavior of the HR and CORDIC_HR neuron model, three error criteria have been used in the form of Eqs. (4) to (6):

4 $$\mathrm{Mean \,Absolute\, Error }\left({\text{MAE}}\right)=\frac{1}{n}\sum_{i=1}^{n}\left|{R}_{CORDIC\, HR\, Neuron}-{R}_{Original\, HR \,Neuron}\right|,$$

5 $${\text{Correlation}}=\frac{cov({R}_{CORDIC\, HR\, Neuron},{R}_{Original \,HR\, Neuron})}{{\sigma }_{{R}_{CORDIC\, HR\, Neuron}}{\sigma }_{Original \,HR\,Neuron}},$$

6 $$\mathrm{Root\, Mean\, Square\, Error }\left({\text{RMSE}}\right)=\sqrt{\frac{\sum_{i=1}^{n}{({R}_{CORDIC\, HR\, Neuron}-{R}_{Original \,HR\, Neuron})}^{2}}{n}}.$$

In Eqs. (4)–(6), ${R}_{Original}$, ${R}_{CORDIC}$ can be any of the variables, *X*, *Y*, and *Z* in the model of HR and CORDIC_HR, respectively. Criteria MAE, Correlation, and RMSE respectively measure the absolute value of the error, statistical dependence, and the mean square of the error between *n* samples of the HR and CORDIC_HR model. In Table 1, these three error criteria for three variables *X*, *Y*, and *Z* are reported for 4 different input current *I*. Columns marked with CORDIC_HR in each error measure are reported in the comparison of the CORDIC_HR neuron vs HR model. Also, columns marked with N_LUT_HR in each error measure are reported in the comparison of the approximate HR neuron based on LUT vs HR model. The N_LUT_HR neuron^22^ is the latest approximation of HR neuron that has been introduced with efficient hardware to replace the original HR neuron model.

**Table 1:** Comparison of the time domain response of CORDIC_HR and N_LUT_HR neurons compared to the original HR neuron model.

Object variable	Mean absolute error		Correlation		Root mean square error	
	CORDIC_HR	N-LUT_HR^22^	CORDIC_HR	N-LUT_HR^22^	CORDIC_HR	N-LUT_HR^22^
X (I = 0.5)	6.57E−04	0.23	0.9999	0.95	0.0026	0.13
X (I = 1.0)	0.0027	0.73	0.9947	0.99	0.0243	1.02
X (I = 1.5)	0.0714	0.23	0.7612	0.99	0.1856	2.02
X (I = 2.0)	0.1008	0.13	0.8702	0.97	0.2055	0.25
Y (I = 0.5)	8.90E−03	0.12	1	0.95	0.0112	0.22
Y (I = 1.0)	0.0256	0.11	0.9989	0.98	0.061	0.42
Y (I = 1.5)	0.2266	0.33	0.9292	0.99	0.4691	1.01
Y (I = 2.0)	0.2613	0.43	0.8253	0.965	0.5326	0.78
Z (I = 0.5)	4.36E−04	0.10	1	0.95	4.63E-04	0.92
Z (I = 1.0)	4.26E−04	0.16	1	0.99	4.74E-04	0.12
Z (I = 1.5)	0.0066	0.63	0.9984	0.98	0.0116	0.32
Z (I = 2.0)	0.0205	0.53	0.9961	0.955	0.0274	0.52

**Figure 5:**
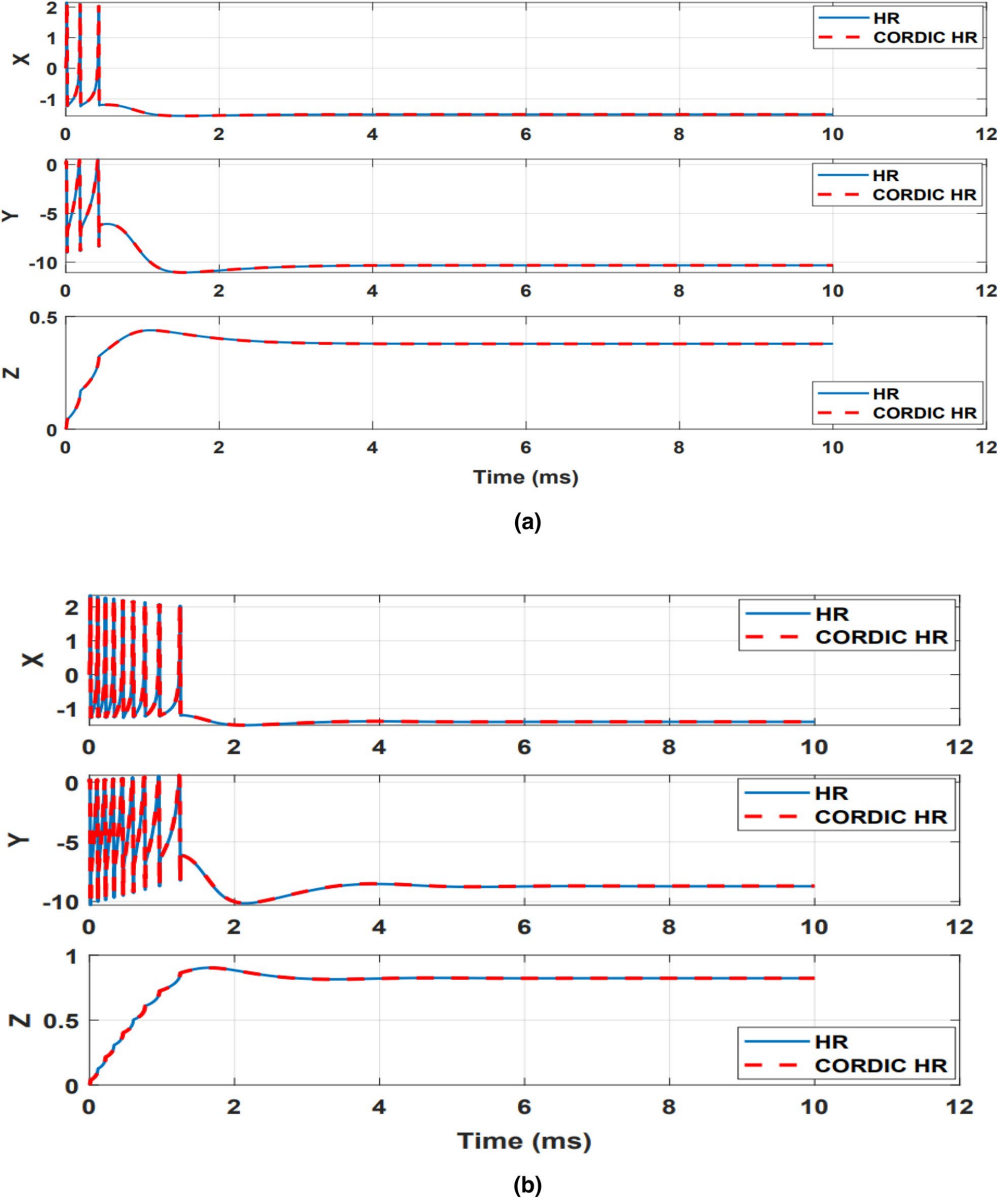

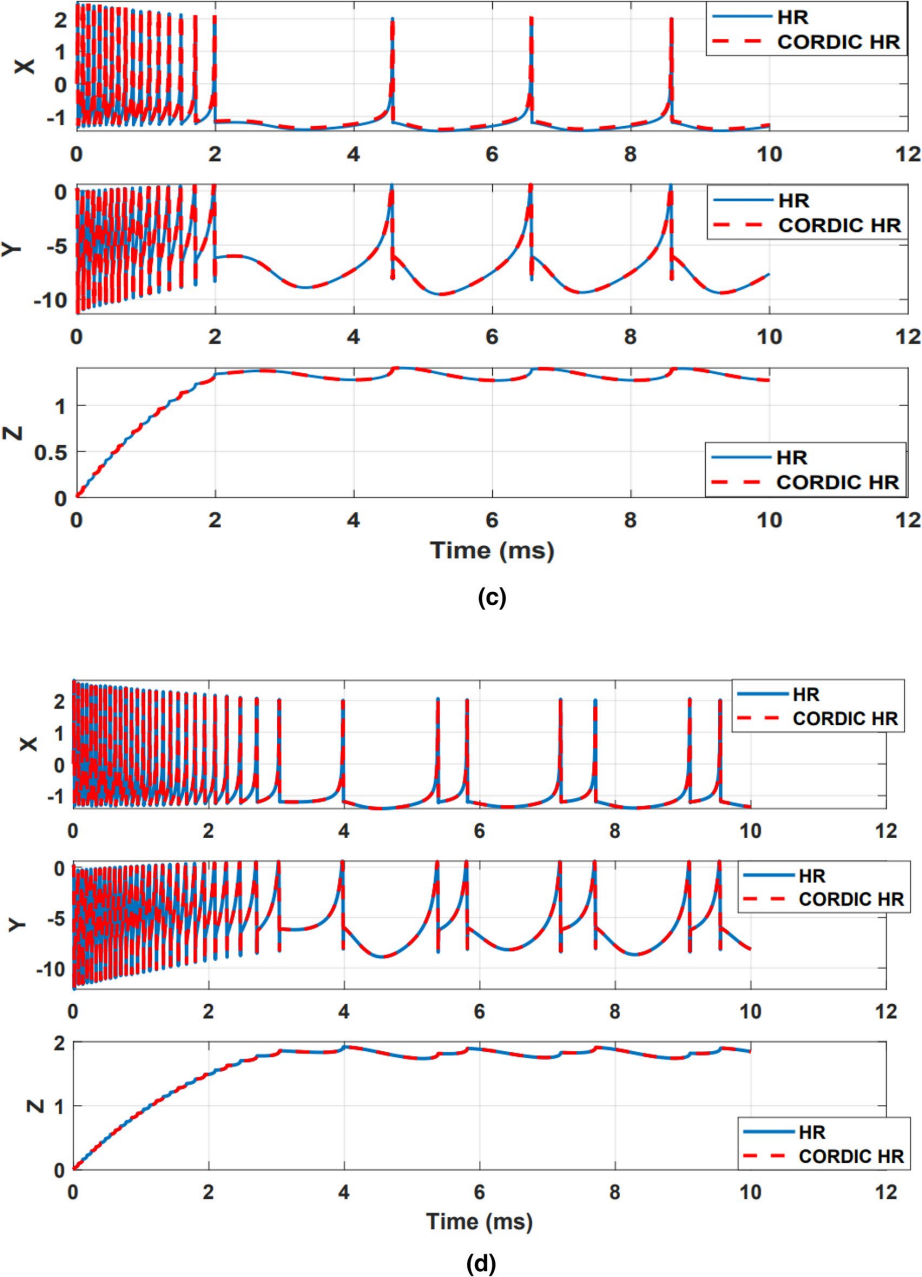
Spiking response of the CORDIC_HR and HR neuron models for different input currents (**a**) I = 0.5, (**b**) I = 1, (**c**) I = 1.5, (**d**) I = 2. In all simulations, r is equal to 0.0021.

As it is evident from the results of Table 1, compared to N_LUT_HR neuron, the proposed CORDIC_HR neuron model follows the behavior of the original HR neuron model in the time domain with much less error. The high accuracy of the proposed CORDIC_HR model in matching to the original HR neuron in the response of the time domain can be seen in Fig. 5, which shows all three variables of the neuron model for different currents.

### Investigating the dynamic behavior of the CORDIC_HR model

**Figure 6:**
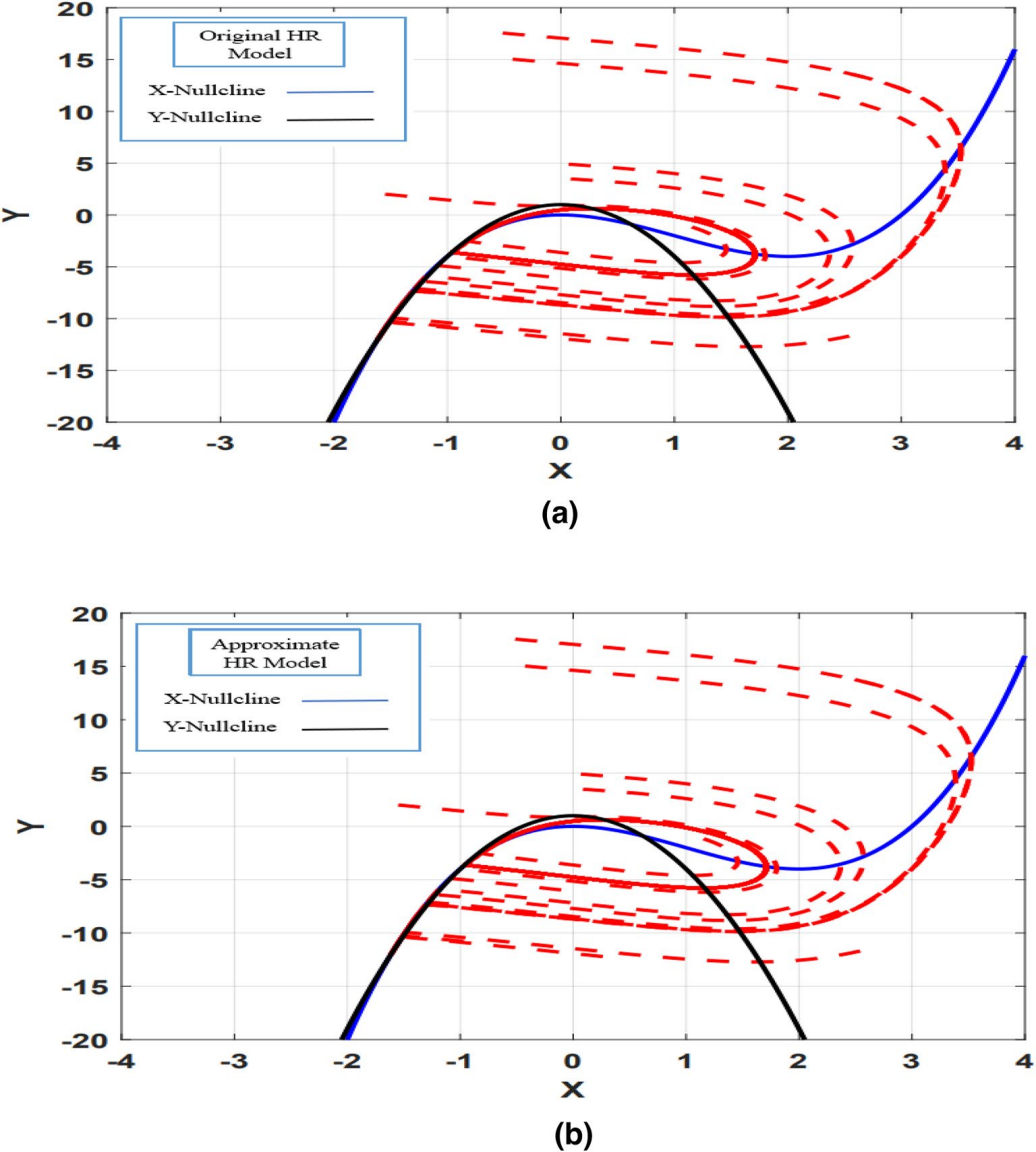
The X–Y nullclines in the HR neuron (**a**) and CORDIC_HR neuron (**b**). As it is evident, the number and type of equilibrium points are the same in the original and proposed models.

**Figure 7:**
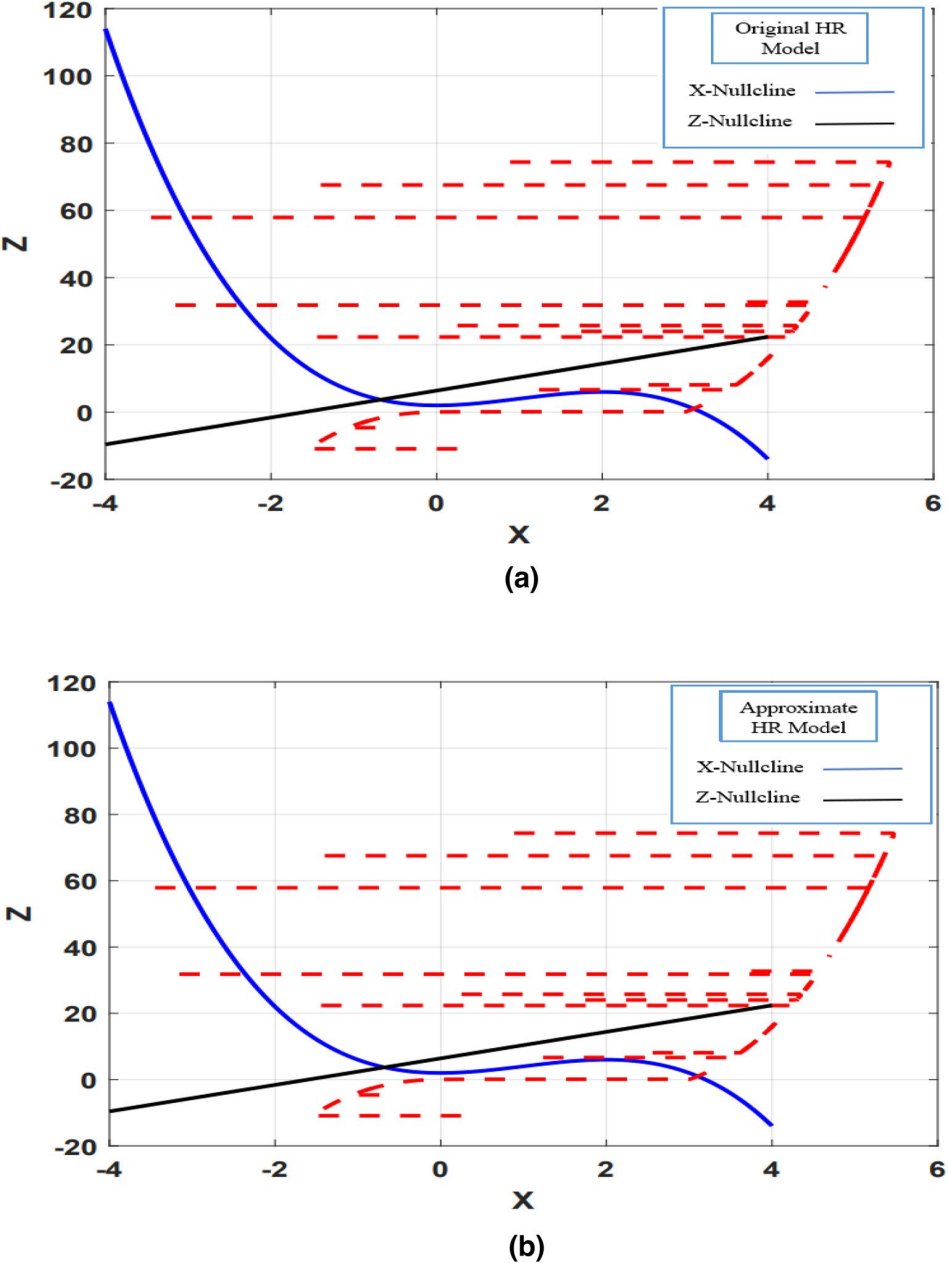
The X–Z nullclines in the HR neuron (**a**) and CORDIC_HR neuron (**b**). There is a complete matching of the behavior of the trajectories in the original and proposed model.

After checking the compatibility of the proposed model with the original model in the time domain, the dynamic behavior matching of the models should also be checked. For this purpose, the behavior of trajectories in nullcline space X–Y and X–Z for the CORDIC_HR and HR models are shown in Figs. 6 and 7. The similarity of the behavior of the trajectories in the nullcline space shows that the original and proposed neuron models have the same equilibrium points in terms of number and type. The number and type of equilibrium points play the most important role in the stability of a dynamic system and it is very important not to change them in the proposed model^9^. The equilibrium points of the dynamic model are equivalent to the collision points of nullclines, which are shown in Figs. 6 and 7, that the equilibrium points of the HR and CORDIC_HR models are the same. Also, the type of equilibrium points can be seen from the behavior of the trajectories in the nullcline space, which according to Fig. 6 (X–Y nullcline space) and Fig. 7 (X–Z nullcline space), there is a complete matching of the behavior of the trajectories for the HR and CORDIC_HR models. In the following, first X-nullcline and Y-nullcline for the HR and CORDIC_HR models have been calculated.

7 $$HR\, Model: \left\{\begin{array}{l}X{\text{-}}nullcline:\,\frac{dX}{dt}=0\\ Y{\text{-}}ullcline:\,\frac{dY}{dt}=0\end{array}\right.\, if\, Z=1 \to \left\{\begin{array}{l}Y={X}^{3}-3{X}^{2}+1-I\\ Y=1-5{X}^{2}\end{array},\right.$$

8 $$CORDIC\_HR\, Model: \left\{\begin{array}{l}X{\text{-}}nullcline:\,\frac{dX}{dt}=0\\ Y{\text{-}}nullcline:\,\frac{dY}{dt}=0\end{array}\right.\, if \,Z=1 \to \left\{\begin{array}{l}Y=CORDIC\_Pow3\_X-3CORDIC\_Pow2\_X+1-I\\ Y=1-5CORDIC\_Pow2\_X\end{array}.\right.$$

In order to show two-dimensional nullcline space (X-nullcline and Y-nullcline) and pay more attention to the movement of trajectories, the third variable Z set to a fixed value. Figure 6 shows the X-nullcline and Y-nullcline in the HR and CORDIC_HR neuron models.

Next, X-nullcline and Z-nullcline for the HR and CORDIC_HR models have been calculated.

9 $$HR\, Model: \left\{\begin{array}{l}X{\text{-}}nullcline:\, \frac{dX}{dt}=0\\ Z{\text{-}}nullcline:\,\frac{dZ}{dt}=0\end{array}\right.\, if \,Y=1 \to \left\{\begin{array}{l}Z=-{X}^{3}+3{X}^{2}+1+I\\ Z=4X+6.4\end{array},\right.$$

10 $$CORDI{C}_{HR}Model: \left\{\begin{array}{l}X{\text{-}}nullcline:\,\frac{dX}{dt}=0\\ Z{\text{-}}nullcline:\,\frac{dZ}{dt}=0\end{array}\right.\, if \,Y=1 \to \left\{\begin{array}{l}Z=-CORDI{C}_{Pow{3}_{X}}+3CORDI{C}_{Pow{2}_{X}}+1+I\\ Z=4X+6.4\end{array}\right..$$

Also, to show two-dimensional nullcline space (X-nullcline and Z-nullcline) and pay more attention to the movement of trajectories, the third variable Y set to a fixed value. Figure 7 shows the original and CORDIC approximation of the X-nullcline and Z-nullcline.

The results presented in Figs. 6 and 7 show that the dynamic characteristics of the HR neuron are fully preserved in CRDIC_HR neuron. Thus, the proposed CORDIC-based approximation, while reduce the hardware cost, can imitate the original neuron model with very high accordance in the time and nullclines space.

### Investigating the phase space behavior of the CORDIC_HR model

**Figure 8:**
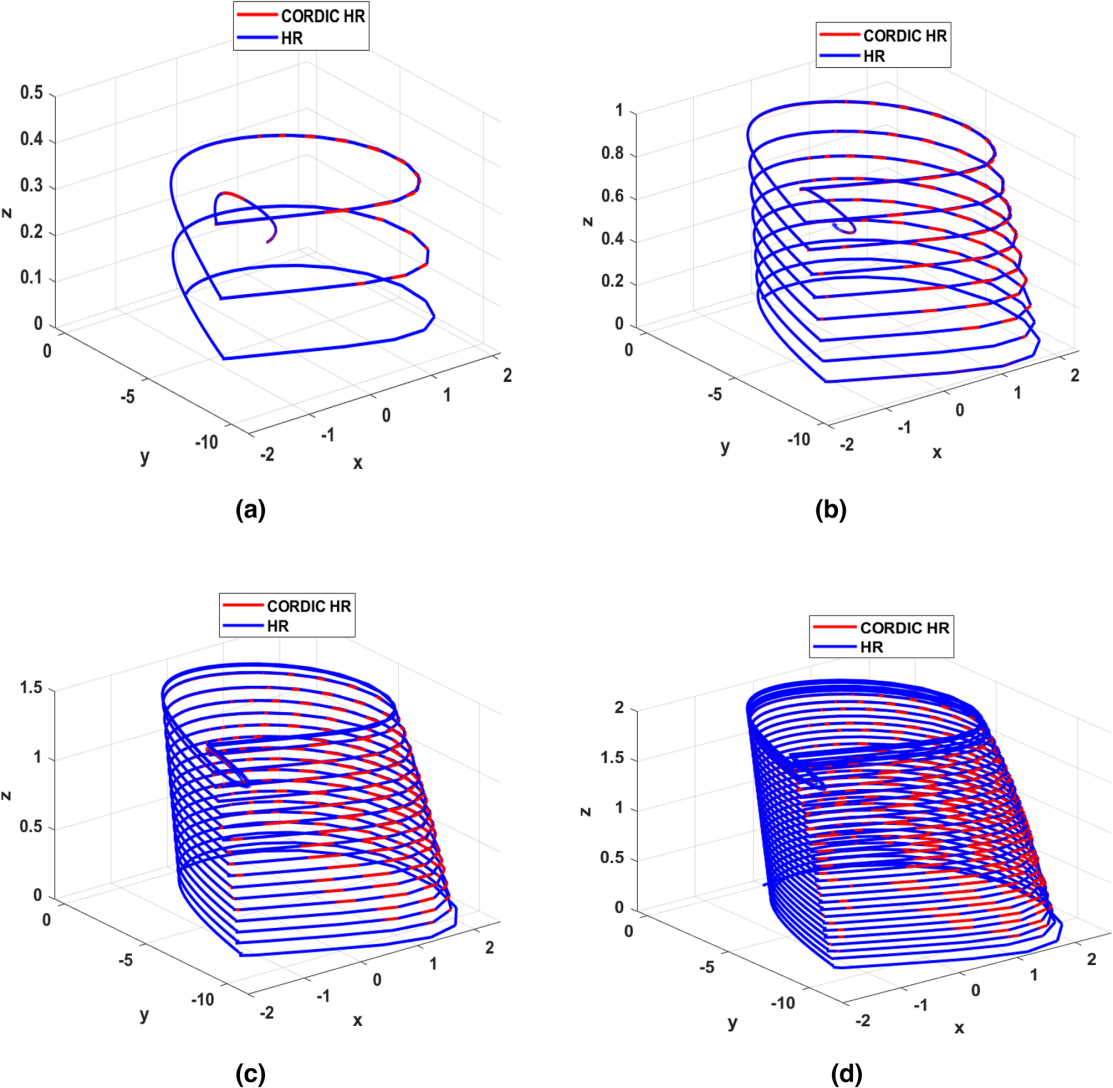
Indicate the phase space of the original and proposed model of HR neuron in blue and red color. The phase space is plotted for different input currents *I*: (**a**) *I* = 0.5, (**b**) *I* = 1, (**c**) *I* = 1.5, (**d**) *I* = 2. In all simulations, *r* is equal to 0.0021.

Phase space analysis is a very important tool in investigating the dynamic behavior of a system. Examining the phase space behavior of the three main variables (*X, Y, Z*) of the original and proposed HR model helps to further validate CORDIC_HR model. In Fig. 8, the phase space for the HR model is drawn in blue color and for the CORDIC_HR model in red color for 4 different input stimulus currents.

As can be seen in Fig. 8, the phase space behavior of the approximate model in different simulation conditions is consistent with the original model. Up to this part, the approximate model has behaved the same as the original model in 3 different analyses. In the next part, a much more comprehensive test has been done on matching the dynamic behavior of two models by analyzing the bifurcation diagram of the original and approximate model of HR neuron.

### Investigating the bifurcation diagram of the CORDIC_HR model

The bifurcation diagram is an essential tool in investigating the complex dynamic behavior of biological models^27^. The complex nonlinear behavior of the CORDIC_HR neuron by changing the system parameters can be numerically analyzed through bifurcation diagram. Analysis of the bifurcation diagram shows that CORDIC_HR neuron has complex dynamic and nonlinear behavior with the change of system parameters.

The ISI (inter-spike interval) is a very important physiological characteristic of neuron behavior. Various encodings have been defined on the spiking response of neurons, in the meantime, temporal coding emphasizes the information transmitted through the interval between spikes in a spike train. Also, many studies^28^ emphasize the transmission of information in the nervous system based on chaotic ISI trail. Pursuant to the sequence of ISIs of neuron, the spiking response pattern of neurons can be divided into two general categories: periodic and non-periodic (chaotic firing pattern)^29^. In the following, the bifurcation diagram of ISI is shown in relation to the change of parameters *I* and *r* for the HR and CORDIC_HR model.

#### The effect of parameter I on the dynamic behavior of the CORDIC_HR model

**Figure 9:**
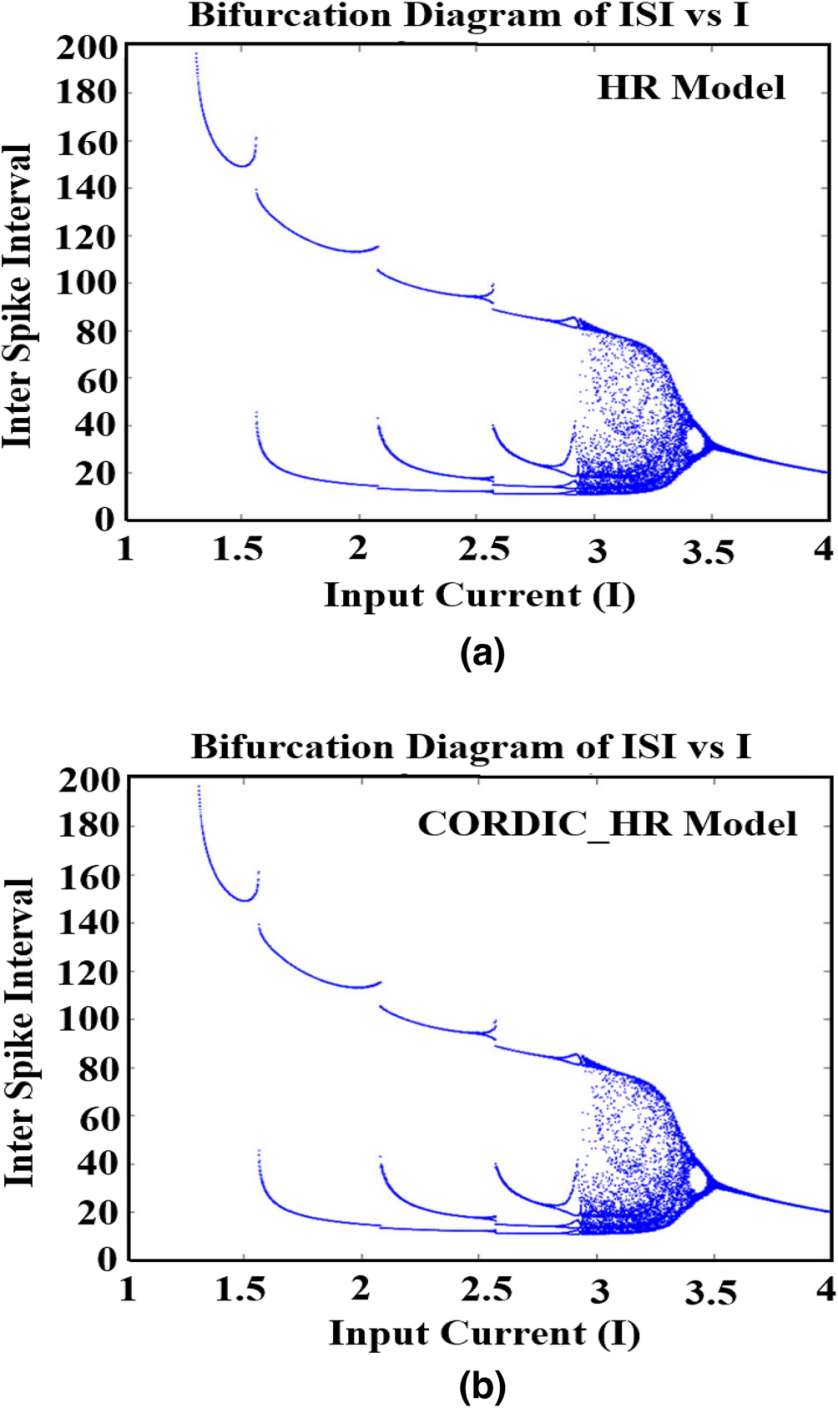
The bifurcation diagram of ISI sequences versus the input current *I* for the HR model^26^ (**a**) and CORDIC_HR model (**b**).

As mentioned, the HR neuron can produce a variety of observable behaviors in a biological neuron. The bifurcation diagram of ISI with respect to the input current as the control parameter that changes from 1 to 4 is shown for the HR model in Fig. 9a and for the CORDIC_HR model in Fig. 9b. In this simulation, the initial variable value (${X}_{0},{Y}_{0},{Z}_{0}$) is considered equal to (0.1, 1, 0.2) and *r* is fixed at 0.005. Figure 9 is an important reference in comparing the dynamic and stability characteristics of HR neuron model with the proposed CORDIC_HR model. Figure 9 shows the correspondence between the behavior of the proposed and the original neuron model in a wide range of input current changes.

By changing the control-parameter *I* from 1, ISIs with periods of 1, 2, 3, and 4 are produced respectively that a period-adding bifurcation phenomenon is observed. By increasing the value of parameter *I* to 3.2, the ISI sequence become unstable and enter the chaotic stage. The interesting behavior is that as the parameter *I* approaches 3.5, the ISI sequence change from chaotic and unstable state to stable state with period 1. According to Fig. 9, it can be concluded that the topology and dynamics of the HR and CORDIC_HR neuron becomes more and more complicated with the increase of parameter *I*, and when *I* reaches the critical value of the system, it returns to a stable state with simple spiking behavior. During the changes of the control parameter *I*, the topological behavior of the system changes from the stable state to the unstable and chaotic state and then to the stable state. The agreement in the bifurcation diagram of the HR and CORDIC_HR model confirms that proposed neuron matches HR neuron with high accuracy.

#### The effect of parameter r on the dynamic behavior of the CORDIC_HR model

**Figure 10:**
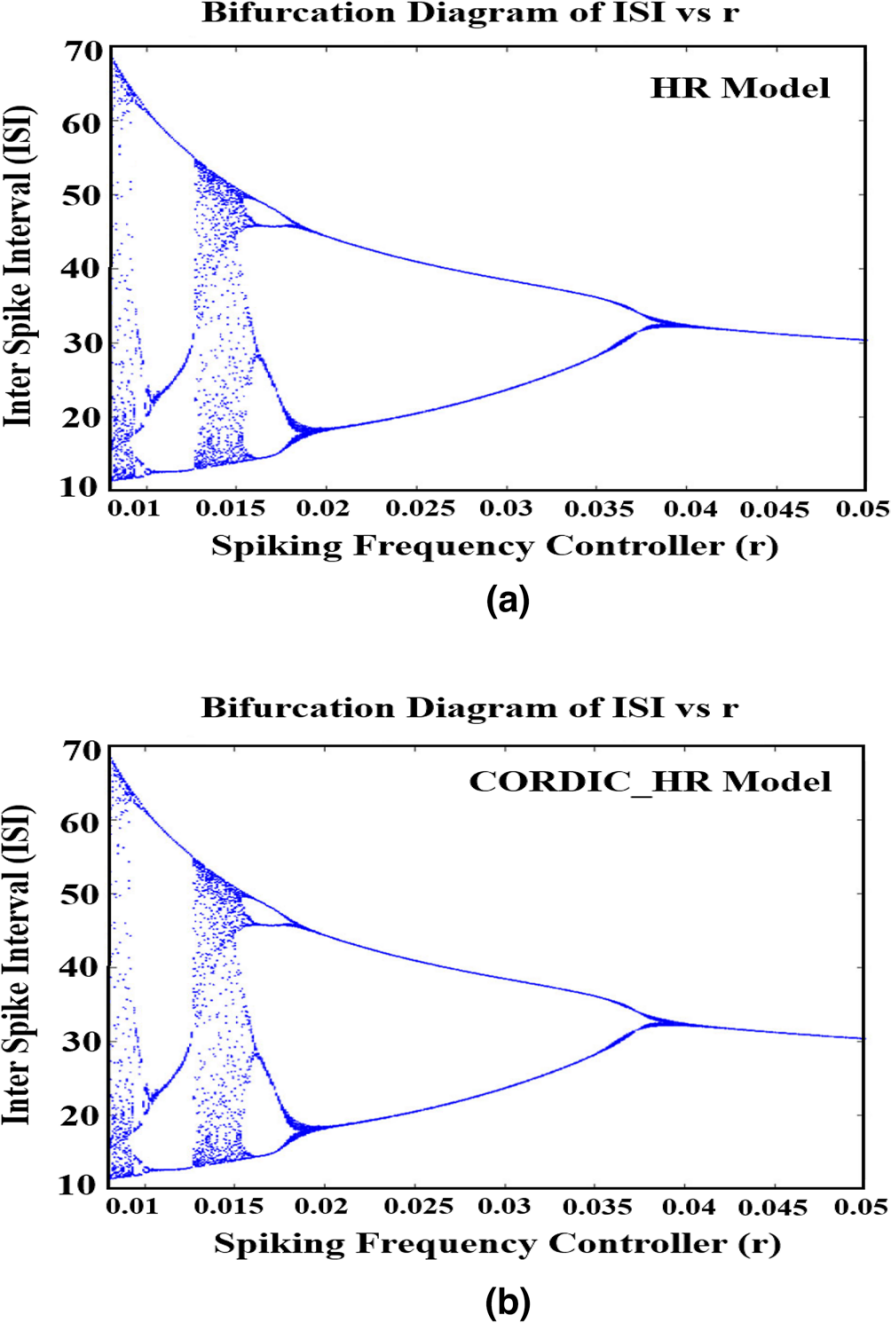
The bifurcation diagram of ISI sequences versus the control parameter *r* for the HR model^26^ (**a**) and CORDIC_HR model (**b**).

In addition to the effect of changing control parameter I on ISI sequence, parameter r is also an important parameter that is equivalent to the accumulation of calcium^30^. For this reason, the bifurcation diagram of ISI sequence of the HR and CORDIC_HR neuron model in relation to changes of control parameter *r* is shown in Fig. 10. In this section, parameter *r* is considered as a control parameter, and by changing it, different spike patterns are produced, and other parameters are considered according to the previous section, and the input current *I* is fixed at a constant value 3.

By changing the control parameter *r* from 0 to 0.05, responses of ISI sequences are produced identical for the HR and CORDIC_HR models and different spiking patterns appear. According to Fig. 10, by changing parameter *r*, the HR neuron shows various spiking behaviors, both in the original and in the approximate neuron model. In fact, HR neuron starts with chaotic and unstable behavior with *r* equal to 0.006 and gradually with increasing *r* enters a stable state with period 4 in *r* equal to 0.007 and period 2 in *r* equal to 0.018 and finally the behavior of period 1 in *r* greater than 0.038. According to Fig. 10, during the changes of the control parameter *r*, the topological behavior of the system changes from the unstable and chaotic state to the stable periodic state. The agreement in the bifurcation diagram of the HR and CORDIC_HR model in Figs. 9 and 10 confirms that proposed neuron matches original HR neuron with high compatibility.

## Large scale network of CRDIC_HR model

**Figure 11:**
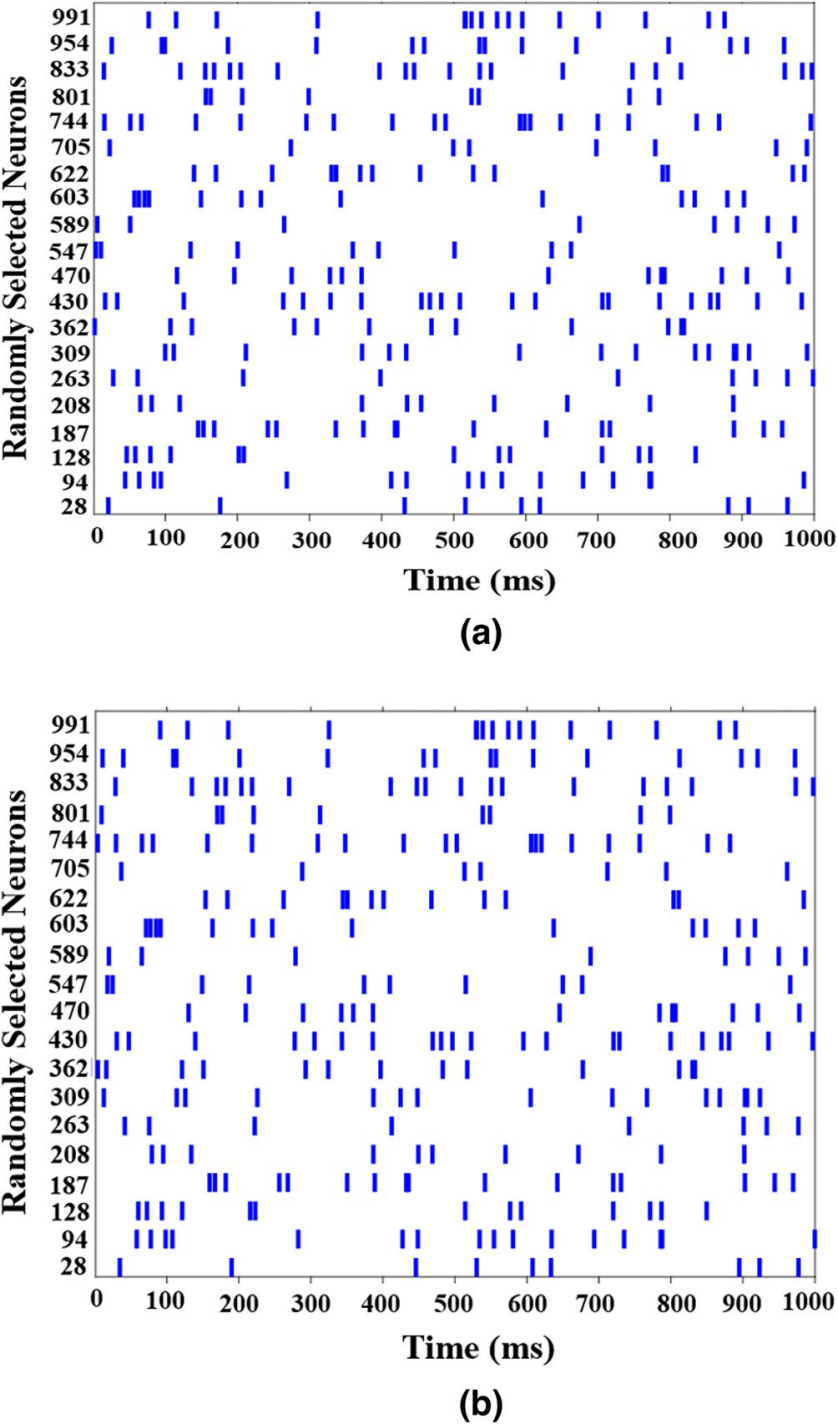
Raster plot of spiking behavior of 20 randomly selected neurons of the HR_network (**a**) and CORDIC_HR_network (**b**).

Since the necessity of providing the CORDIC_HR neuron model with a lower computational cost than the HR model was considered in the possibility of implementing an efficient large-scale network of CORDIC_HR neurons in the hardware, the collective behavior of CORDIC_HR neurons should also be investigated. Thus, two populations of 1000 neurons are developed, which one using HR neurons (HR_network) and the other using CORDIC_HR neurons (CORDIC_HR_network). In both networks, 80% of the neurons are excitatory and 20% are inhibitory, and neurons are randomly connected with a probability of 0.2. Therefore, each neuron is randomly connected to approximately 200 other neurons, and in general there are approximately 200,000 synapses in each network. Synapses in both networks are considered as weighted connections with a constant weight of 1. In case that input stimulation current *I* is set to 0.5 for both networks, Fig. 11 shows the raster plot of spiking behavior of 20 randomly selected neurons of the HR_network (a) and CORDIC_HR_network (b).

According to Fig. 11, it is evident that the collective behavior of the HR and CORDIC_HR neurons in networks with random connections of 1000 neurons are completely compatible. Therefore, if we can design the digital circuit of the CORDIC_HR neuron with the lowest cost and area that can be scaled to large scale network in hardware, CORDIC_HR neuron can reproduce the biological behavior of the HR neuron with high accuracy.

## Digital circuit design

Considering that the HR model has nonlinear terms and creates the need for a multiplier in the hardware implementation, the multiplier less hardware implementation of the proposed CORDIC_HR model is discussed in this section. In the CORDIC HR model, the nonlinear terms have been replaced by the CORDIC approximation, and the digital circuit of the CORDIC_HR model can only be implemented using addition, subtraction, and shift. The approximation of non-linear terms causes the CORDIC_HR hardware implementation to have less consumption resources and subsequently less area, and higher working frequency compared to HR model.

### Considerations in the selection of parameters and bit-width

In this design, the multiplier is not used to multiply the fixed parameters in the variables, and shift and addition are used instead. Therefore, the selection of parameters such as $\Delta t$ has been done in such a way that delta multiplication can be done only with shift.

In this design, due to the reduction of hardware cost, the numbers have been used in the form of fixed-point registers. In each part of the design, to reduce the consumption of hardware resources, the minimum bit length is considered for fixed point calculations for each variable. According to the range of changes of variables *X, Y*, and *Z* in Fig. 5, 4 bits are needed for representation of these main variables in the HR and CORDIC_HR model, but considering that the value of these variables change during the shift and addition calculations, the bit-width of 15 is considered, which one bit is reserved as sign bit.

### Discretization of differential equations

The differential equations of the proposed CORDIC_HR model are continuous equations and these equations must be discretized for digital design. There are different discretization methods including Runge–Kutta and Euler with different orders, which the first order Euler method is used due to the simplicity and accuracy. The discretized equations of variables *X, Y, Z* are given in Eq. (11).

11 $$\left\{\begin{array}{l}X\left[i+1\right]=X\left[i\right]+\Delta t\{Y\left[i\right]+3\times CORDIC\_POW\_2 X[i]-CORDIC\_POW\_3 X[i]-Z[i]+I\}\\ Y\left[i+1\right]=Y\left[i\right]+\Delta t\{1-5\times CORDIC\_POW\_2 X[i]-Y[i]\}\\ Z\left[i+1\right]=Z\left[i\right]+\Delta t\{4\times r\times X\left[i\right]+6.4\times r-r\times Z\left[i\right]\}\end{array}.\right.$$

In Eq. (11), $\Delta t$ is the discretization step of the equations and considered to be 1/256 so that it can be easily multiplied by only 9 times shift right.

### Scheduling diagrams

**Figure 12:**
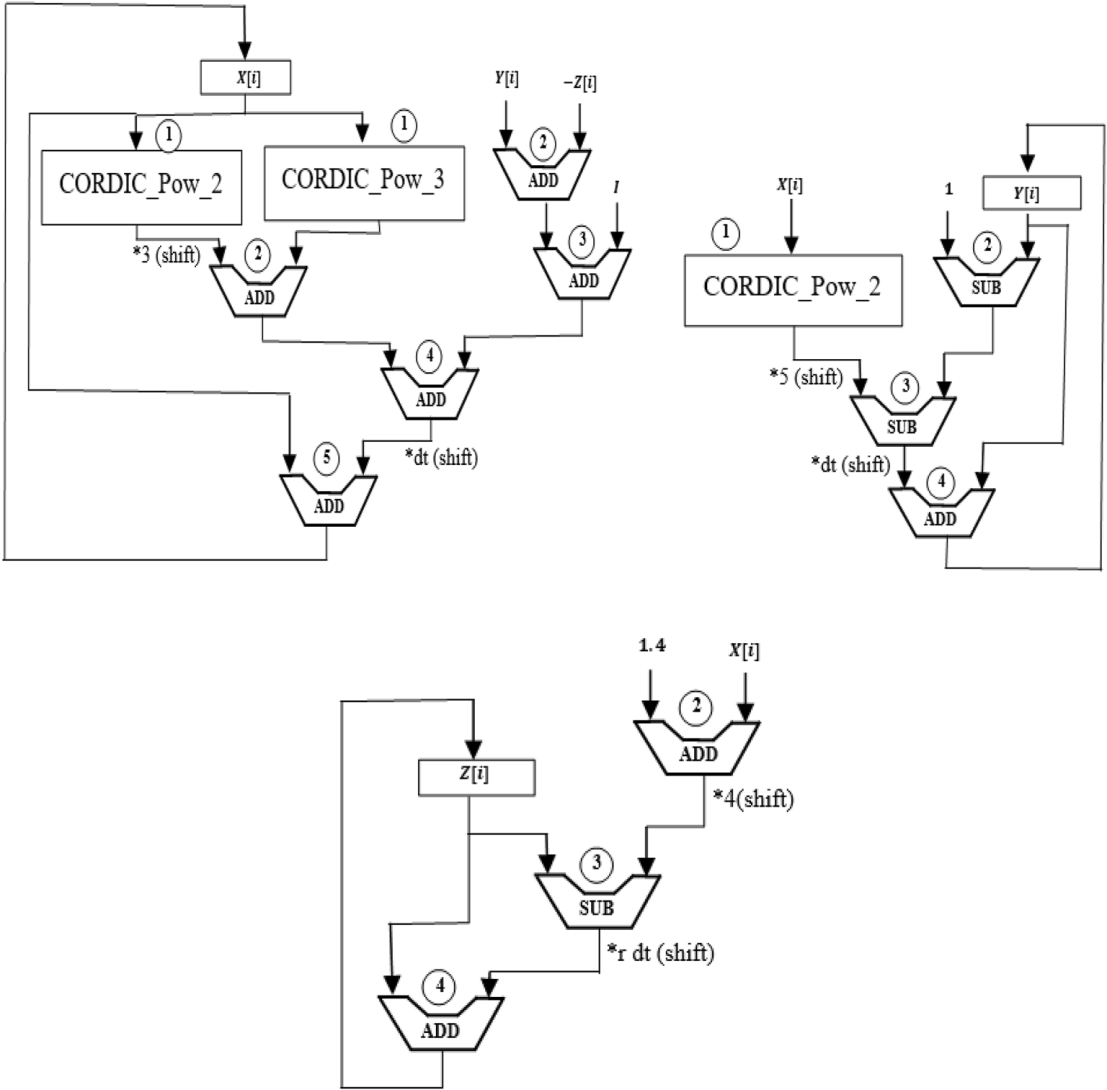
Scheduling diagram of *X, Y, Z* equations of CORDIC_HR neuron model.

Figure 12 shows the scheduling diagram of equations *X, Y, Z*. In this design, non-linear terms have been removed and instead of them, CORDIC blocks of power 2 and power 3 have been placed, and instead of using a multiplier, shift and addition operations have been replaced.

According to the scheduling diagram, the proposed CORDIC_HR model can be implemented on hardware without using a multiplier.

### Overall structure of CORDIC_HR digital circuit

**Figure 13:**
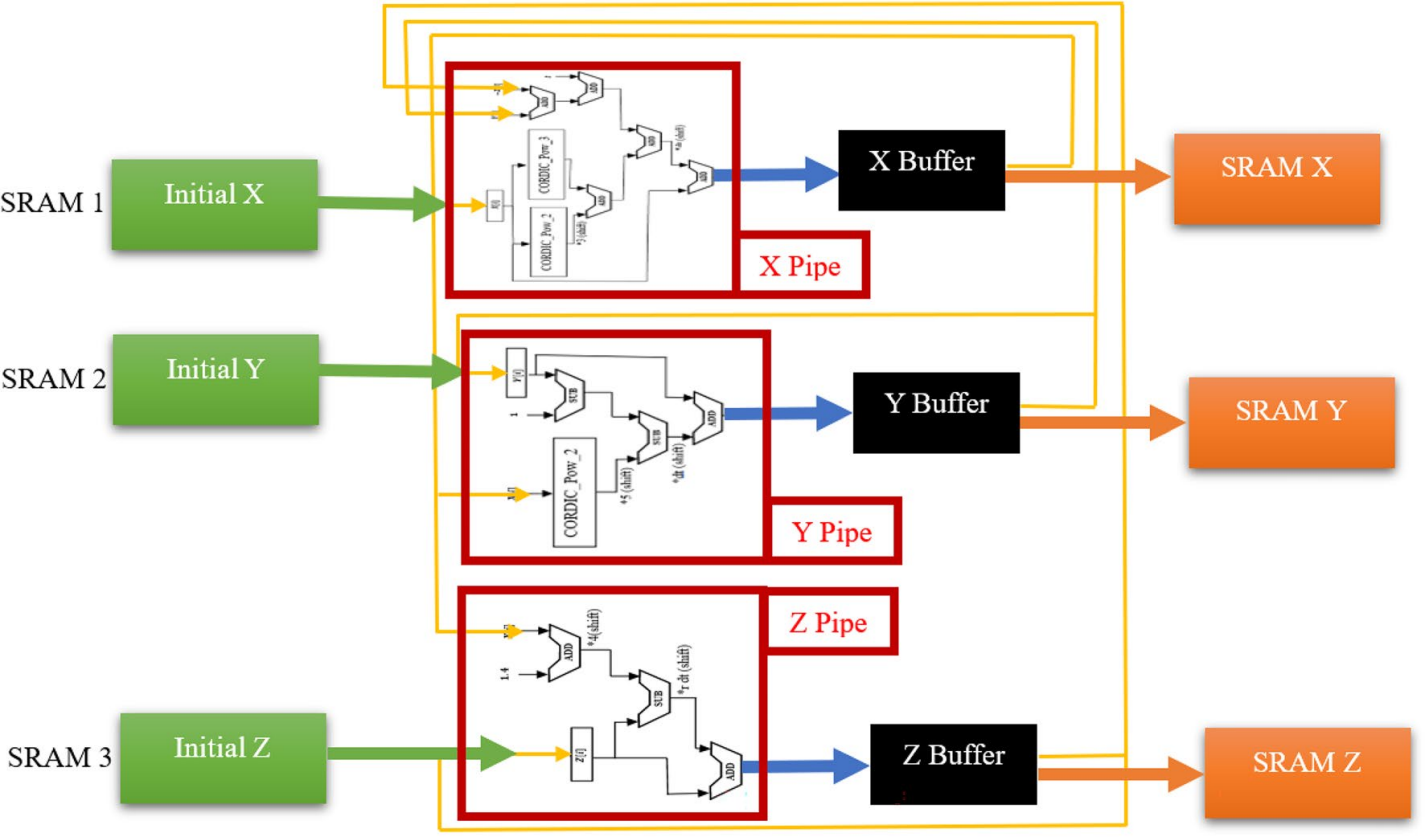
Overall structure of CORDIC_HR digital circuit.

Overall structure of CORDIC_HR digital circuit can be designed as Fig. 13. As emphasized in the previous sections, the proposed CORDIC_HR neuron model with a very low error compared to HR model, has provided the possibility of multiplier-less implementation on the hardware. On the other hand, according to the scheduling diagram, the digital design of the CORDIC_HR neuron is possible only by using low-cost adder, subtractor and shift blocks. The overall architecture is designed in such a way that the constant parameters and initial values of *X, Y, Z* are called from the corresponding SRAMs and applied to the digital blocks *X, Y, Z*. Pipes *X, Y, Z* are considered to speed up the execution of neural computations, although the hardware cost increases slightly with the parallelization considered.

### Hardware cost comparison

In the proposed CORDIC_HR model, to reduce the hardware cost, non-linear terms have been replaced with low-cost computing blocks based on CORDIC. Accordingly, in this section, the necessity of presenting the proposed model is clarified by comparing the hardware cost in the implementation of HR and CORDIC_HR neuron model. In Table 2, a comparison of the resources used in the digital design of the HR and CORDIC_HR models is presented. According to the results reported in Table 2, the proposed model has the minimum hardware cost and the highest frequency compared to the original HR model and other presented models. On the other hand, considering that the proposed CORDIC_HR model compared to previous studies has very low error in imitating the behavior of the HR neuron from various aspects, the proposed model can be a suitable option for implementing a large-scale neural network on hardware.

**Table 2:** Resource utilization of FPGA in digital designs of HR neuron model.

Digital HR model	Slice Flip Flop	4-in-LUT	Speed (MHz)	Multiplier	Adder	Subtractor
Original HR	852	268	25	8	7	6
Kazemi et al.^31^	284	831	87.7	2	2	2
Hayati et al.^32^	412	659	81.2	0	N/A	N/A
Cai et al.^16^	469	N/A	139	0	N/A	N/A
Esteban et al.^33^	1173	2172	94.23	104	N/A	N/A
CORDIC_HR neuron	285	224	110	0	4	3

The CORDIC is a synchronous circuit block. The CORDIC block ${X}^{2}$ require 31 clock cycle to complete a single CORDIC operation with 11 iterations. Thus, The CORDIC block ${X}^{3}$ require 31 × 2 = 62 clock cycle to complete a single operation. In Table 2, the speed is reported for the most critical path in the circuit synthesized and implemented by ISE. Therefore, the frequency presented in Table 2 is for the maximum delay, which actually shows the nominal frequency of the proposed circuit.

## Discussion

Hardware implementation of neurons has been an attractive topic in recent years because it enables the implementation of bio-inspired processing systems in the form of large-scale neural networks on hardware. Today’s computers are very powerful in terms of computing power, but it is very important to design processors with the ability to reproduce the responses of the nervous system that can improve the cognitive ability of today’s machines. High-level cognitive capabilities, which are the weak point of today’s smart machines, are created by the collective behavior of the neurons of the nervous system. For this reason, by designing hardware neurons with minimum consumption resources, it is possible to create a population of neurons on the hardware and finally processors with the close functionality as the nervous system with higher cognitive capabilities than today’s machines. The purpose of this paper in the first stage was to provide an efficient digital design of a biological neuron model such as HR neuron, so that the approximate model (CORDIC_HR) can mimic the behavior of the original model (HR) with high accuracy. In the next step, through the simulation of a network of 1000 neurons, it was shown that the collective behavior of CORDIC_HR neurons is akin to the collective behavior of HR neurons. Up to this point, considering that the proposed CORDIC_HR neuron follows the behavior of the original HR neuron well and has a collective behavior alike the HR neurons it is time to test the performance of the CORDIC_HR neuron in image processing applications.

**Figure 14:**
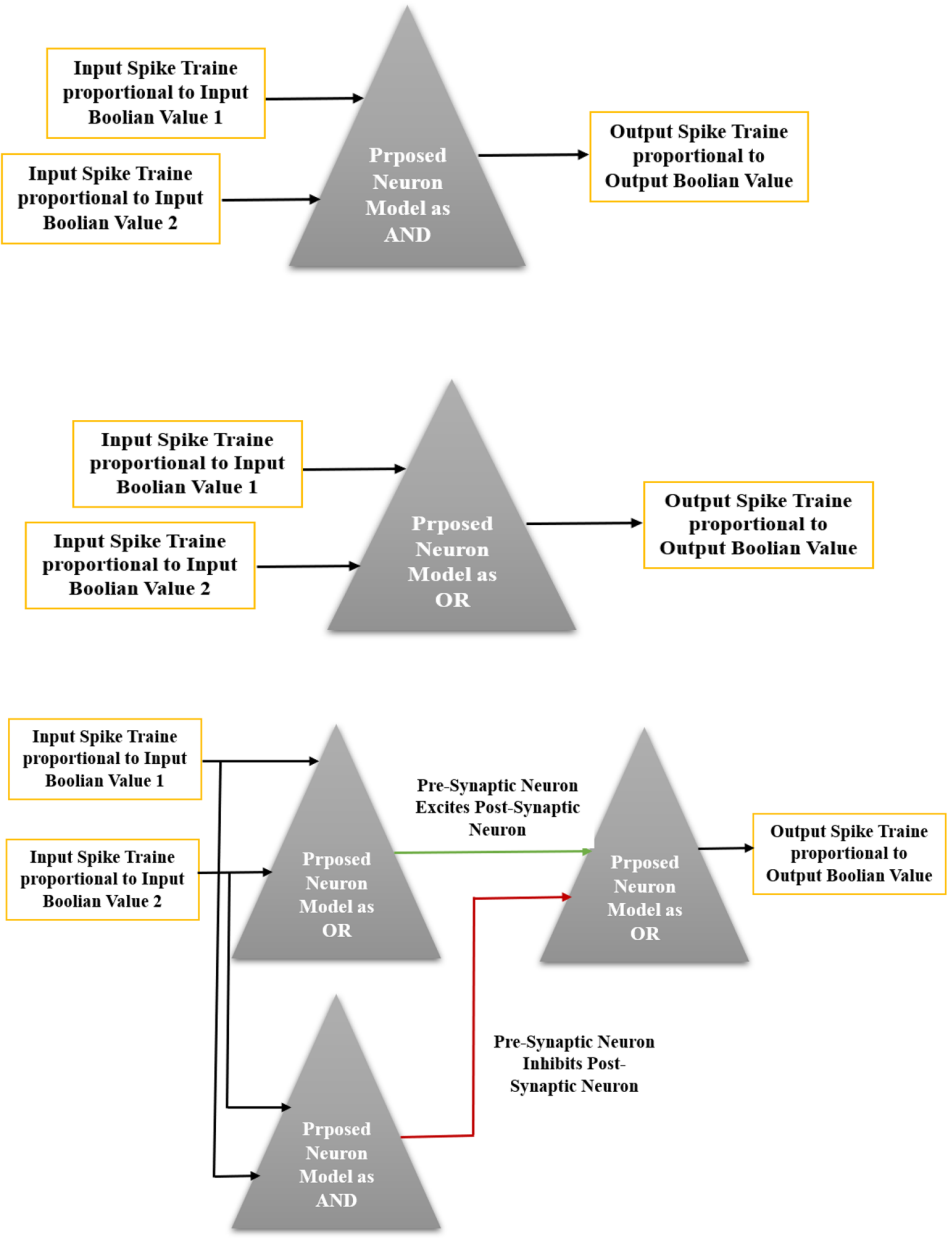
The CORDIC_HR neuron used to make SFGs (spiking AND, OR, and NOT gates).

Previous studies introduce spiking frequency gates (SFGs) which can emulate the performance of Boolean gates such as AND, OR, NOT using the frequency of spike trains^34^. Considering that Inter Spike Interval or in other words spike frequency is of high importance in the transmission of information in biological systems, this coding has been used in the mapping of spike information to Boolean values. In the spiking frequency gates, to map Boolean values to spike information or vice versa, to map spike train to Boolean values, a frequency range is considered, so that spike frequencies less than 5 Hz represent zero Boolean value and spike frequencies greater than 5 Hz represent Boolean value one. With the approach akin the previous study^34^ which make SFGs based on LIF neuron, spiking gates were designed using the proposed CORDIC_HR neuron model according to Fig. 14. It is necessary to mention that the synaptic equations in the production of SFGs are according to the previous study^34^ and only the LIF neuron model has been replaced with the CORDIC_HR neuron.

Considering that spiking AND, OR gates have two inputs and one output, in Table 3, 4 states that may happen in their inputs are listed. Inputs of AND, OR gates can be one of 4 states (0,0), (0,1), (1,0), and (1,1) and subsequently inputs of spiking AND, OR gates can be one of 4 states (Spike train with a frequency of 0 to 5, Spike train with a frequency of 0 to 5), (Spike train with a frequency of 0 to 5, Spike train with a frequency higher than 5), (Spike train with a frequency higher than 5, Spike train with a frequency of 0 to 5), (Spike train with a frequency higher than 5, Spike train with a frequency higher than 5). Table 3 shows the performance of spiking frequency gates AND, OR based on CORDIC_HR neuron by applying different input spike trains that simulate four possible input states. The results of Table 3 show that by considering the spike train with a frequency below 5 Hz as a logic zero and a spike train with a frequency above 5 Hz as a logic one, the proposed spiking frequency gates AND, OR based on CORDIC_HR neuron answer correctly and the performance of the logic gates AND, OR is implemented in the form of spiking gates.

**Table 3:** Spiking frequency gates AND, OR based on CORDIC_HR neuron.

Input spike trains {Boolean value}		Output spike trains {Boolean value}	
Spike train frequency in input 1	Spike train frequency in input 2	Output spike train frequency in AND	Output spike train frequency in OR
[0–5) Hz {0}	[0–5) Hz {0}	[0–5) Hz {0}	[0–5] Hz {0}
[0–5) Hz {0}	[5–20] Hz {1}	[0–5) Hz {0}	[5–20] Hz {1}
[5–20] Hz {1}	[0–5) Hz {0}	[0–5) Hz {0}	[5–20] Hz {1}
[5–20] Hz {1}	[5–20] Hz {1}	[5–20] Hz {1}	[5–20] Hz {1}

With the same scenario, the performance of spiking frequency gate NOT based on CORDIC_HR neuron is listed in Table 4, which shows the match of spiking gate NOT performance with its logical counterpart.

**Table 4:** Spiking frequency gate NOT based on CORDIC_HR neuron.

	Spike train frequency in input {Boolean value}	Spike train frequency in output {Boolean value}
NOT	[0–5) Hz {0}	[5–20] Hz {1}
	[5–20] Hz {1}	[0–5) Hz {0}

In the next sections, spiking frequency gates AND, OR, NOT based on CORDIC_HR neuron are used in the design of spiking networks for edge detection, image magnification, and noise removal^35^. So far, various spiking networks have been proposed for machine vision applications such as pattern recognition^36^, noise removal^37^, edge detection^38^. The main difference between the previous spiking networks and the spiking networks that are discussed in the rest of this paper is that the networks based on spiking gates of CORDIC_HR neuron can perform processing operations on the image without going through the training and learning process.

### Spiking edge detector platform based on CORDIC_HR neuron

In Eq. (12), a morphological filter called CL filter is introduced^35,39^, which is used for image edge detection. As it is evident in Eq. (12), the edge detection operation can be done using AND, NOT.

12 $$f\left(i,j\right)=g\left(i,j\right) CAND CNOT\left[g\left(i-1,j\right)CANDg\left(i,j-1\right)CANDg\left(i+1,j\right)CANDg\left(i,j+1\right)\right].$$

**Figure 15:**
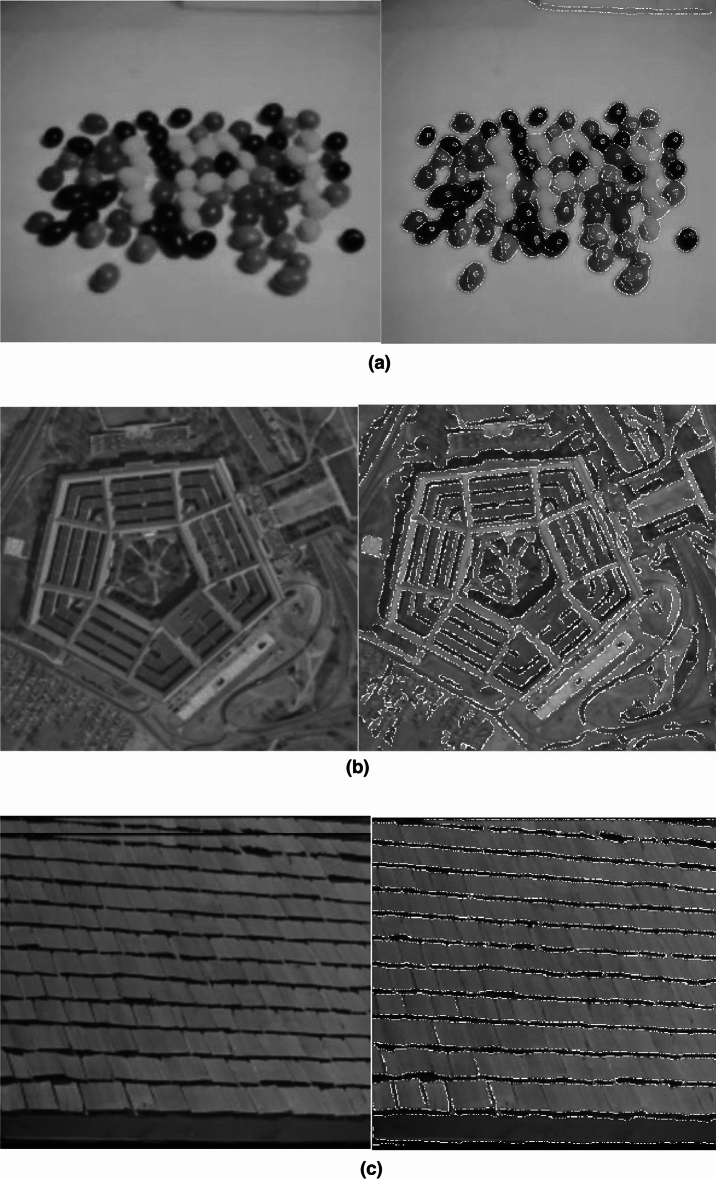
Spiking edge detector based on CORDIC_HR model.

By replacing the logic gates AND, NOT with spiking frequency gates AND, NOT designed with the CORDIC_HR neuron, the spiking edge detector based on CORDIC_HR neuron can be developed. In order to check the performance of the spiking edge detector based on CORDIC_HR model, examples of edge detection with this spiking platform are given in Fig. 15. The strength of the spiking edge detector based on CORDIC_HR model is that it does not require training with large data and engineering of feature extraction from images for edge detection.

### Spiking image magnification platform based on CORDIC_HR neuron

In Eq. (13), a CL filter is introduced, which is used for image magnification^35,39^. As it is evident in Eq. (13), this operation can be done using OR gate.

13 $$f\left(i,j\right)={g}_{1}\left(i,j\right)COR {g}_{1}\left(i,j+1\right)COR {g}_{1}\left(i,j+2\right)COR {g}_{1}\left(i+1,j\right)COR {g}_{1}\left(i+1,j+1\right)COR {g}_{1}\left(i+1,j+2\right) COR {g}_{1}\left(i+2,j\right) COR {g}_{1}\left(i+2,j+1\right) COR {g}_{1}\left(i+2,j+2\right).$$

**Figure 16:**
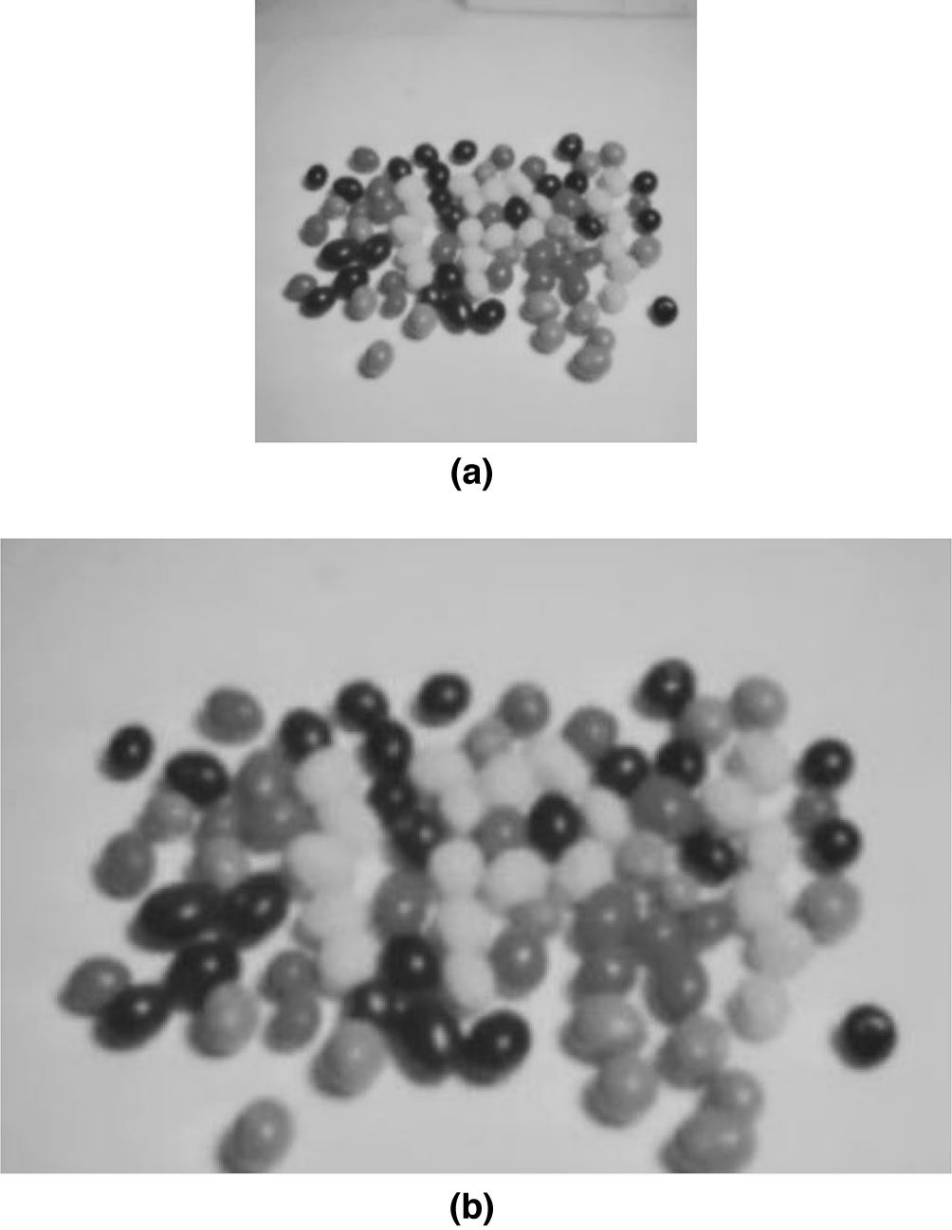
Spiking image magnification based on CORDIC_HR model. (**a**) Is the input image and (**b**) is the magnified image with scale 3.

By replacing the logic gates in Eq. (13) with spiking gates designed with the CORDIC_HR neuron model, the spiking image magnification platform is obtained. To confirm the performance of the spiking image magnification based on CORDIC_HR model, example of its operation with a magnification factor of 3 is given in Fig. 16.

### Spiking noise removal platform based on CORDIC_HR neuron

**Figure 17:**
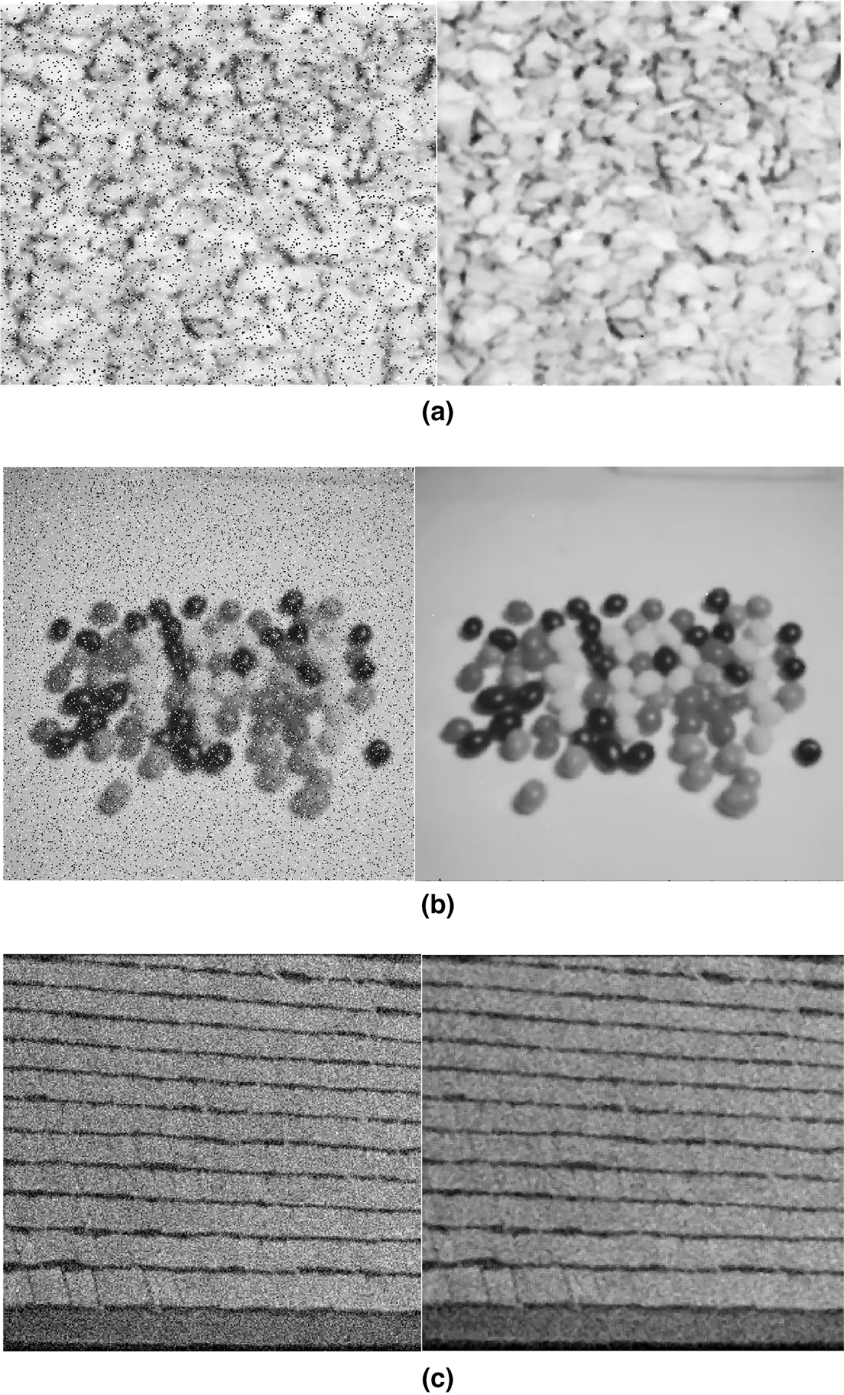
Spiking noise removal platform based on CORDIC_HR model. (**a,b**) Indicate salt and pepper noise removal performance and (**c**) shows gaussian noise removal performance.

In Eq. (14), a CL filter is introduced, which is used for noise removal^35,39^. By replacing the logic gates AND, OR with spiking frequency gates AND, OR designed with the CORDIC_HR neuron, the spiking noise removal platform based on CORDIC_HR neuron can be developed. To investigate the performance of the spiking noise removal platform based on CORDIC_HR model, examples of noise removal with this spiking platform are given in Fig. 17.

14 $$f\left(i,j\right)=\left[g\left(i,j\right)CAND g\left(i+1,j\right)CAND g\left(i,j+1\right)\right]COR\left[g\left(i,j\right)CAND g\left(i,j+1\right)CAND g\left(i,j-1\right)\right]COR\left[g\left(i,j\right)CAND g\left(i+1,j\right)CAND g\left(i-1,j\right)\right]COR\left[g\left(i,j\right)CAND g\left(i,j-1\right)CAND g\left(i+1,j\right)\right]COR\left[g\left(i,j-1\right)CAND g\left(i+1,j\right)CAND g\left(i,j+1\right)\right]COR\left[g\left(i,j-1\right)CAND g\left(i,j+1\right)CAND g\left(i-1,j\right)\right] COR\left[g\left(i+1,j\right)CAND g\left(i,j+1\right)CAND g(i-1,j)\right]COR\left[g\left(i,j-1\right)CAND g\left(i-1,j\right)CAND g(i+1,j)\right]COR\left[g\left(i,j-1\right)CAND g\left(i-1,j\right)CAND g(i,j)\right].$$

For the purpose of more accurately examine the performance of the spiking noise removal platform based on CORDIC_HR model, in Table 5 a quantitative comparison of the performance of the proposed platform in comparison with other noise removal methods for removing noise of Poisson and salt & pepper has been reported.

**Table 5:** A quantitative comparison of the performance of the spiking noise removal platform based on CORDIC_HR model in comparison with other noise removal methods.

Noise removal method	Poisson noise with $\lambda =1$		Salt and pepper noise with 10% noise probability	
	MSE (mean squared error)	PSNR (peak signal-to-noise ratio (in dB))	MSE	PSNR
Arithmetic filter^40^	114.31	27.54	27.04	33.81
Geometric filter^40^	24.46	34.24	30.54	33.28
Harmonic filter^40^	31.15	33.19	34.03	32.81
Contra-harmonic filter^40^	252.15	24.11	252.12	24.11
Median filter^40^	19.79	35.16	9.29	38.44
Max and min filter^40^	79.63	29.11	94.15	28.39
Mid-point filter^40^	16.24	36.02	15.95	36.10
Spiking noise removal network^35^	20.8	33.40	9.79	36.50
Spiking noise removal platform based on CORDIC_HR model	20.3	34.1	9.2	36.8

The processing power of the human brain while consuming low power is a question that has been the focus of researchers’ studies for years. Neuromorphic systems are the manifestation of circuits that are compatible with neural system computations and their hardware design is done efficiently^41^. The efficient digital design of CORDIC_HR neuron in this paper can be used as a neuromorphic platform with low power consumption in machine vision applications.

Finally, work innovations can be categorized as follows:

- Proposed CORDIC_HR neuron which imitates the complex nonlinear behavior of HR neuron with high accuracy while it requires less resources for hardware implementation.
- The proposed CORDIC_HR neuron has the same collective behavior as the HR neuron in a large-scale neural interaction.
- The proposed neuron performs processing capabilities such as noise removal, image magnification and edge detection better than previous spiking platforms without need to go through the training process.

In fact, the reason for choosing the CORDIC_HR neuron for edge detection is to show the computational ability of the proposed model in image processing.

## Conclusion

In this paper, an efficient digital circuit for the HR neuron model was presented, which was the digital implementation circuit of the proposed CORDIC_HR neuron. In the CORDIC_HR model, the nonlinear terms of the HR model have been replaced by efficient CORDIC blocks. Based on Table 2, the presented circuit of the CORDIC_HR neuron compared to the previous studies in the digital implementation of HR neuron consumes less resources and subsequently occupies less area and has a higher working frequency. To check the accuracy of the performance of the proposed CORDIC_HR model in imitating the responses of the original HR model, comparing the response of the two models in the time domain, the movement of the trajectories in the nullcline space, and comparing the behavior of their phase space have been reported and the high compatibility of the CORDIC_HR model from the original one was confirmed. In addition, the complex nonlinear behavior of the CORDIC_HR neuron compared to HR model by changing the system parameters was analyzed through bifurcation diagram and the high accordance of two models was confirmed. Since the necessity of providing the CORDIC_HR neuron model with a lower computational cost than the HR model was considered in the possibility of implementing an efficient large-scale network in the hardware, the collective behavior of CORDIC_HR neurons was investigated and high accordance with original model was obtained. Finally, spiking frequency gates AND, OR, NOT were presented based on the proposed neuron, which led to the design of spiking edge detector, noise removal and image magnification platform based on CORDIC_HR neuron model. The proposed spiking platforms based on spiking gates of CORDIC_HR neuron can perform processing operations on the image with acceptable accuracy without going through the learning process. In general, the main contribution of the paper is in presenting an efficient hardware model, which consumes less hardware resources, follows the behavior of the original model with high accuracy, and performs noise removal and edge detection process on image with acceptable accuracy. Therefore, the efficient digital design of CORDIC_HR neuron in this paper can be used as a neuromorphic platform with low hardware cost in machine vision applications.

## Data Availability

Data would be available through corresponding author with reasonable request.
